# A Comprehensive Physicochemical Analysis Focusing on the Characterization and Stability of Valsartan Silver Nano-Conjugates

**DOI:** 10.3390/ijms27020582

**Published:** 2026-01-06

**Authors:** Abdul Qadir, Khwaja Suleman Hasan, Khair Bux, Khwaja Ali Hasan, Aamir Jalil, Asad Khan Tanoli, Khwaja Akbar Hasan, Shahida Naz, Muhammad Kashif, Nuzhat Fatima Zaidi, Ayesha Khan, Zeeshan Vohra, Herwig Ralf, Shama Qaiser

**Affiliations:** 1 Molecular and Structural Biochemistry Research Unit, Department of Biochemistry, University of Karachi, Karachi 75270, Pakistanshahidanaz19891@gmail.com (S.N.); 2 Department of Chemistry, University of Karachi, Karachi 75270, Pakistan; hksulaeman@gmail.com (K.S.H.); akbar_hasan@outlook.com (K.A.H.); 3 Department of Biosciences, Faculty of Life Sciences, Shaheed Zulfiqar Ali Bhutto Institute of Science and Technology (SZABIST), Karachi 75600, Pakistan; 4 MAHQ Biological Research Centre, University of Karachi, Karachi 75270, Pakistan; 5 Department of Pharmaceutical Sciences, Bahauddin Zakaria University, Multan 60800, Pakistan; 6 Institute of Chemistry, Shah Abdul Latif University Khairpur, Khairpur 66020, Pakistan; 7 Clinical Biochemistry and Hematology Research Laboratory, Department of Biochemistry, Federal Urdu University, Karachi 75300, Pakistan; 8 Department of Biochemistry, University of Karachi, Karachi 75270, Pakistan; 9 International Center for Chemical and Biological Sciences (ICCBS), H.E.J. Research Institute of Chemistry, University of Karachi, Karachi 75270, Pakistan; 10 Laboratories PD Dr. R. Herwig, 80337 Munich, Germany; 11 Heimerer-College, 10000 Pristina, Kosovo; 12 Aga Khan University Hospital, Karachi 74800, Pakistan

**Keywords:** valsartan, nano-conjugation, solubility, *Mangifera indica*, differential scanning calorimetry, hemocompatibility

## Abstract

Valsartan (Val)—a lipophilic non-peptide angiotensin II type 1 receptor antagonist—is highly effective against hypertension and displaying limited solubility in water (3.08 μg/mL), thereby resulting in low oral bioavailability (23%). The limited water solubility of antihypertensive drugs can pose a challenge, particularly for rapid and precise administration. Herein, we synthesize and characterize valsartan-containing silver nanoparticles (Val-AgNPs) using *Mangifera indica* leaf extracts. The physicochemical, structural, thermal, and pharmacological properties of these nano-conjugates were established through various analytical and structural tools. The spectral shifts in both UV-visible and FTIR analyses indicate a successful interaction between the valsartan molecule and the silver nanoparticles. The resulting nano-conjugates are spherical and within the size range of 30–60 nm as revealed in scanning electron-EDS and atomic force micrographs. The log-normal distribution of valsartan-loaded nanoparticles, with a size range of 30 to 60 nm and a mode of 54 nm, indicates a narrow, monodisperse, and highly uniform particle size distribution. This is a favorable characteristic for drug delivery systems, as it leads to enhanced bioavailability and a consistent performance. Dynamic Light Scattering (DLS) analysis of the Val-AgNPs indicates a polydisperse sample with a tendency toward aggregation, resulting in larger effective sizes in the suspension compared to individual nanoparticles. The accompanying decrease in zeta potential (to −19.5 mV) and conductivity further supports the idea that the surface chemistry and stability of the nanoparticles changed after conjugation. Differential scanning calorimetry (DSC) demonstrated the melting onset of the valsartan component at 113.99 °C. The size-dependent densification of the silver nanoparticles at 286.24 °C correspond to a size range of 40–60 nm, showing a significant melting point depression compared to bulk silver due to nanoscale effects. The shift in Rf for pure valsartan to Val-AgNPs suggests that the interaction with the AgNPs alters the compound’s overall polarity and/or its interaction with the stationary phase, complimented in HPTLC and HPLC analysis. The stability and offloading behavior of Val-AgNPs was observed at pH 6–10 and in 40% and 80% MeOH. In addition, Val-AgNPs did not reveal hemolysis or significant alterations in blood cell indices, confirming the safety of the nano-conjugates for biological application. In conclusion, these findings provide a comprehensive characterization of Val-AgNPs, highlighting their potential for improved drug delivery applications.

## 1. Introduction

The oral administration of drugs is considered as one of the most convenient therapeutic approaches. However, 40% of pharmaceutical products are poorly water-soluble [1,2]. Drugs possessing low water solubility in body fluids are challenging for drug developers because they are difficult to deliver effectively through oral administration [3]. Therefore, the pharmaceutical scientists put forward different physical and chemical methods to increase solubility and stability in the aqueous phase. Overcoming the limited solubility, nanomaterials and nanoformulations like metallic nanoparticles (MNPs), magnetic nanoparticles, polymeric nanoparticles, carbon nanotubes, liposomes, dendrimers, and quantum dots have revolutionized the drug administration [4,5].

There are numerous chemical and physical methods that have been devised for the synthesis of metal nanoparticles, MNPs, and most of these are not cost-effective and produce toxic byproducts [6,7,8]. In addition, many of the reactants used in the chemical synthesis are potentially unsafe for medical applications [9]. On the other hand, physical methods need expensive equipment and a higher temperature and pressure due to the massive consumption of energy. Reductive biosynthesis is considered a simple, reproducible, non-toxic, environment-friendly, cost-effective, and more sustainable process for the production of NPs in bulk [10]. The reductive biosynthesis of NP_S_, through plant extracts, is believed to be an environmentally friendly and unexpensive method. The plant extracts are potential sources of phytochemicals, including polyols, terpenoids, and polyphenols, catalyzing the reduction and capping of metal ions. The green chemistry is widely adopted for the synthesis of different nanoparticles such as gold, silver, zinc oxide, and iron quite easily [11,12].

Valsartan (Val)—a lipophilic non-peptide angiotensin II type 1 receptor antagonist—is highly effective against hypertension and displays limited solubility in water (3.08 μg/mL), thereby resulting in low oral bioavailability (23%). The limited water solubility of antihypertensive drugs can pose a challenge, particularly for rapid and precise administration. In addition, the management of blood pressure in intensive care units could be challenging in critically ill patients. It is generally believed that oral medication can be considered if there is no target organ damage mediated by hypertension. However, urgent blood pressure reduction treatment is required for concurrent events such as acute ischemic or hemorrhagic stroke [13]. It is widely acknowledged in pharmaceutical research that drug-loaded nanocarriers and nanoparticles display higher dermal penetration and absorption compared to conventional formulations. This enhanced efficacy in transdermal drug delivery is due to several unique properties of nanomaterials [14,15,16,17].

Ensuring the safe and effective delivery of this sparingly water-soluble drug is crucial. This study focuses on improving valsartan’s solubility and absorption with the help of nanocarriers. Herein, we report valsartan-containing silver nanoparticles (Val-AgNPs), which were synthesized using reduction and capping methods with *Mangifera indica* leaf extracts. The physicochemical, structural, thermal, and pharmacological properties of these nano-conjugates were established through various analytical and structural tools. The silver nano-conjugates of valsartan might find their way for dermal application and enhanced drug delivery and might be helpful in the management of hypertension.

## 2. Results

### 2.1. Spectrophotometric Characterization of Mangifera indica Leaf Extract, AgNPs, and Val-AgNPs

**Figure 1:**
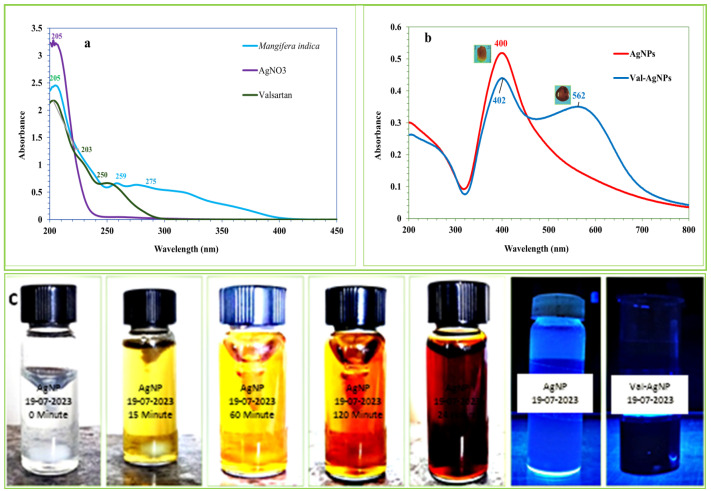
(**a**) UV-Vis absorption spectra of *Mangifera indica*, AgNO_3_, and Valsartan. (**b**) UV-Vis absorption spectral analysis and surface properties of AgNPs and Val-AgNPs synthesized through reductive biosynthesis. (**c**) Color transitions and UV-absorption properties of AgNPs and valsartan silver nano-conjugates at different time intervals.

The UV-Vis absorption spectra of *Mangifera indica* leaf extract in Figure 1a shows the characteristic absorption of light at 259 nm and 275 nm. The absorption of light in this region may correspond to the presence of flavonoids compounds, which typically consist of two major bands, band I (300–380 nm) and band II (240–295 nm), where the former engenders a yellow color; in some flavonoids, the absorption tails to 400–450 nm [18]. The polyphenolic compounds act as reducing and capping agents in the synthesis of silver nanoparticles (AgNPs) and valsartan-loaded silver nanoparticles (Val-AgNPs [19,20,21]. The aqueous solution of silver nitrate AgNO_3_ exhibits maximum absorption (λmax at 205 nm) in the far-UV region. However, the methanolic solution of valsartan showed a characteristic absorption at 249 nm [22].

In Figure 1b, the UV-Vis spectrum of AgNPs exhibited a peak absorbance at 400 nm, falling within the standard range of the surface plasmon resonance (SPR) band of 400–550 nm and the phenomenon is because of the reverberating of light waves with free electrons in silver nanoparticles [23,24]. However, the spectrum of Val-AgNPs revealed plasma resonance peaks along with a bathochromic shift at λmax 402 nm and noticeable hypochromic effect differing from the absorption maxima of standalone valsartan (249 nm) and AgNPs (400 nm). In addition, Val-AgNPs present a broader red shift with the amplitude of the through being measured at 562 nm. This shift might result due to the attachment of valsartan onto the surface of metallic silver. Therefore, increased conjugation may decrease the energy gap between the molecule’s highest occupied molecular orbital (HOMO) and its lowest unoccupied molecular orbital (LUMO), requiring less energy for an electron to transition to an excited state. The AgNPs indicate the color transition from transparent to iron brown during 0–24 h of bio-reductive synthesis and particle stabilization (Figure 1b,c) [25,26]. UV-visible spectroscopy revealed plasma resonance peaks of Val-AgNPs at 402 and 562 nm, differing from the absorption maxima of standalone valsartan (250 nm) and AgNPs (400 nm) in water and methanol solutions. In Val-AgNPs, the bathochromic shifts result in the complexation of AgNPs with valsartan. When a colloidal solution of AgNPs is illuminated, the electrons on the surface of the nanoparticles oscillate at a specific frequency, resonating with the incident light. This absorption of light causes the solution to appear a specific brown color typical for silver nanoparticles. In addition, AgNPs showed blue illumination when irradiated with 253 nm UV-light. On the other hand, Val-AgNPs appeared as a blood red color and absorbed UV-light of 253 nm (Figure 1b,c). In addition, Val-AgNPs showed hydrophobic properties on the glass surface in contrast to AgNPs (Figure 1b).

### 2.2. FTIR Characterization

**Figure 2:**
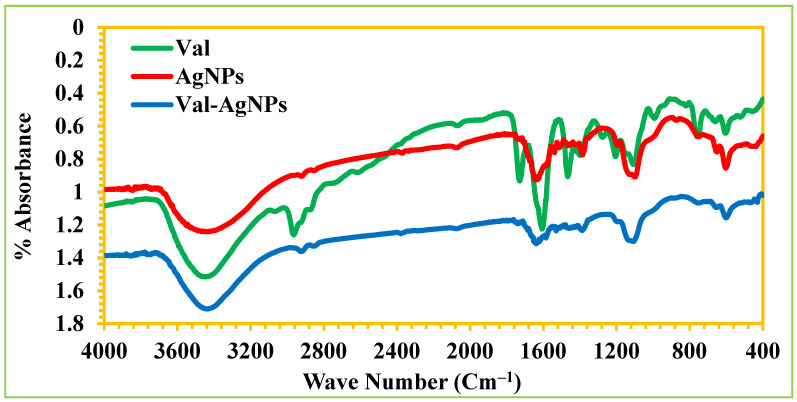
FTIR spectra presenting the functional groups for valsartan, reducing, and capping agents that were characterized onto the surface of AgNPs and Val-AgNPs.

The FTIR spectra analyzed in potassium bromide disks showed the characteristic functional groups, i.e., N–H, C=N, C=O, C–O, and C-N at wavenumbers of 3460.30, 2964.59, 1606.62, 1205.51, and 1107.14 cm^−1^ in valsartan (Figure 2). However, a spectral shift and an increase in the percent absorbance were observed for Val-AgNPs (3439.08, 2922.16, 1631.78, 1192.01, and 1107.14 cm^−1^), respectively (Figure 2). In the baseline-corrected infrared spectra, the intense, clear, and proportionate peaks were selected at 1606 cm^−1^ and 3460 cm^−1^, corresponding to an amide IR spectrum presenting the functional groups for valsartan [27,28] characterized by a strong C=O stretch in the 1600–1680 cm^−1^ range and N–H stretches between 3100 and 3500 cm^−1^ (Figure 2). However, an increase in the C=O stretch frequency of 1631 cm^−1^ and decrease in the N–H stretching of 3439 cm^−1^ was observed in Val-AgNPs (Figure 2). This change in the absorption of functional groups might result due to the attachment of valsartan on the surface of AgNPs [29]. The FTIR spectroscopy identified the functional groups assocaited with biomolecules in the *Mangifera indica* leaf extract responsible for the capping and stabilization of the AgNPs (Figure 2). The FTIR spectrum of AgNPs shows a characteristic bond absorption peak at 3429.43 cm^−1^, which corresponds to the O–H stretching due to the presence of phenolic and carboxylic acid containing phytochemicals in plant extract [30]. Furthermore, the peaks at 2924.09 cm^−1^, 1631.78 cm^−1^, 1450 cm^−1^, 1195 cm^−1^_,_ and 1099.43 cm^−1^ indicate the stretching of C–H, C=C, N–H, C–O, and C=O and reveal the existence of alkane, conjugated alkane, amide, and alcohol in the plant extract [31]. The band at 1384.89 cm^−1^ could be related to the CH_2_ symmetric bending modes of methyl groups of carboxylates. *Mangifera indica* leaf is a rich source of terpenoids, flavonoids, and lignin. These compounds contain various functional groups, like carboxyl, hydroxyl, ketones, and aldehyde [32,33]. The changes in vibrational frequencies determined by FTIR spectroscopy show the characteristic difference between AgNPs and Val-AgNPs, which strengthen the loading of valsartan on the surface of silver nanoparticle and the phytoconstituents of *Mangifera indica* leaf extract, reducing the Ag^+^ to Ag^0^ in bioreductive synthesis, considered as an environmentally friendly and cost-effective method for nanoparticle production [30].

### 2.3. SEM and EDS Analysis

**Figure 3:**
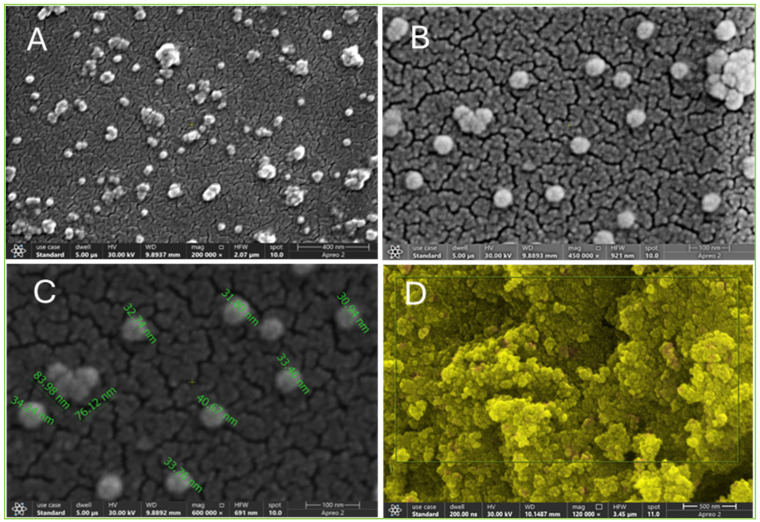
(**A**) Scanning electron micrographs of Val-AgNPs illustrating the morphology, particle size, and distribution visualized at 200,000×. (**B**) Val-AgNPs visualized at 450,000× resolution. (**C**) Illustrating the average distribution and particle size of Val-AgNPs observed at size 600,000×. (**D**) Elemental mapping of Val-AgNP in air-dried material).

The electron micrographs in Figure 3 illustrate the globular-shape Val-AgNPs present in large numbers in the formulation (Figure 3A,B). The average size of singlet particles was in the range of 30 to 40 nm (Figure 3C). However, di, tri, tetra, and octameric forms were also characterized in the electron micrographs. In addition, the larger agglomerates had assembled into globular rosettes (Figure 3B). However, the tetrameric Val-AgNPs has an average area of 76 to 83 nm (Figure 3C). The appearance of agglomerates occurred as a result of sample preparation in the drying step or might be due to the hydrophobic interactions of valsartan, which results in a larger surface-area-to-volume ratio that tends to aggregate to reduce surface energy [34].

**Figure 4:**
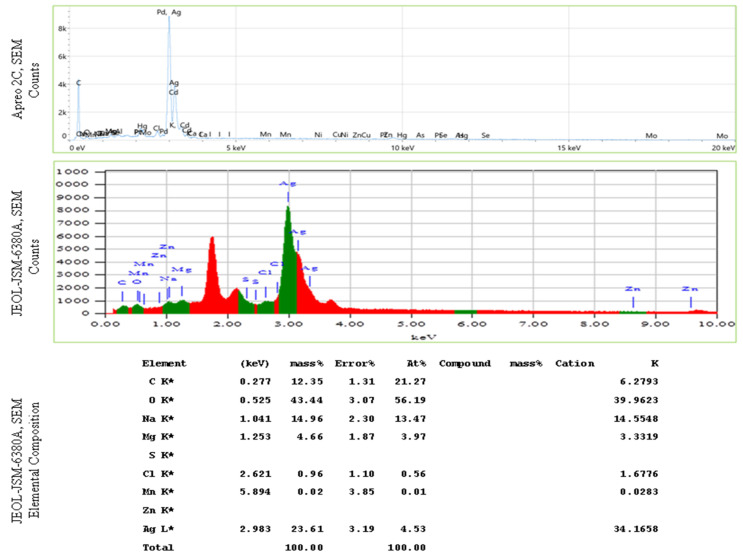
Energy-dispersive spectroscopy (EDS) pattern of Val-AgNPs illustrating the elemental mapping in dispersed smears. The ‘K’ indicates that the X-ray was produced by an electron transition involving the K-shell (the innermost electron shell), and the asterisk (*) typically signifies an artifact peak or a potential peak overlap.

**Figure 5:**
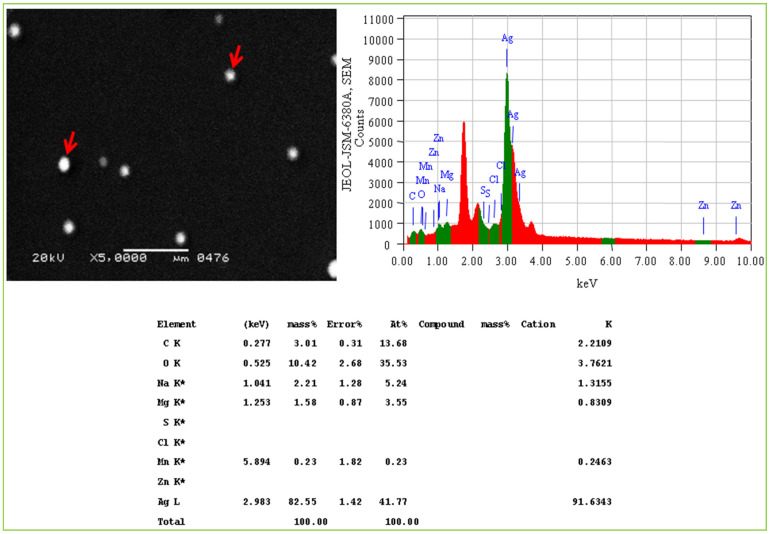
Scanning electron micrographs of AgNPs illustrating the morphology, particle size, distribution, indicated by arrow head (→) and elemental mapping of AgNPs in dispersed smears. The ‘K’ indicates that the X-ray was produced by an electron transition involving the K-shell (the innermost electron shell), and the asterisk (*) typically signifies an artifact peak or a potential peak overlap.

The elemental composition of Val-AgNPs with 500 nm spatial resolution along with the high-resolution information of the sample surface morphology within the same scanned area is indicated in Figure 3D. Acquisition parameters for the elemental analysis indicate the total number of counts, 337,338, the average count rate was 11 266 cps estimated for 30 s, and the data were obtained at an acceleration voltage of 30 kV. The scans obtained through Apreo 2C scanning electron microscope (Theromo Scientific, Waltham, MA, USA) in powdered Val-AgNPs consisted of silver (in the highest percentage), followed by carbon, oxygen, and nitrogen (Figure 4 and Table 1). However, the elemental analysis of Val-AgNPs using the JEOL-JSM-6380A scanning electron microscope (JEOL Ltd., Akishima, Tokyo, Japan) revealed an abundance of oxygen, followed by a silver, sodium, and carbon element (Figure 4). Figure 5 illustrates spherical AgNPs with an average size range of 28–30 nm. The EDS spectra showed a higher percentage of silver, followed by oxygen, carbon, and sodium. The presence of these elements in the AgNPs most likely originated from polyphenolic compounds. AgNPs synthesized using plant extracts contained a higher percentage of oxygen and carbon, suggesting that a higher amount of phytocompounds, primarily phenolic compounds, bound to the nanoparticles’ surface [33,35,36].

### 2.4. AFM Analysis

**Figure 6:**
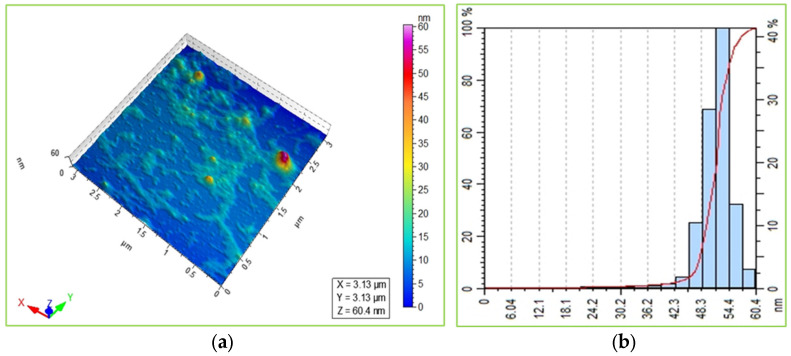
Atomic force microscopic images of Val-AgNPs. Three-dimensional surface morphology and particle distribution of Val-AgNPs (**a**). Particle diameter histogram of Val-AgNPs showing the line plotted corresponds to fit using a log normal distribution (**b**).

The lateral (x, y) and a vertical (z) resolution image of an atomic force microscope in Figure 6a showed the oval-shaped mono and aggregated Val-AgNPs [37]. The log normal distribution of the particle size shows that the valsartan-loaded nanoparticles were ranging from 30 to 60 nm, along with a higher distribution of 54 nm particles in the solution, as indicated in Figure 6a [38,39]. This distribution of the biological synthesis of silver nanoparticles was ideally characterized in a previous study [25].

### 2.5. Zeta Potential Measurements and Particle Size Analysis

**Figure 7:**
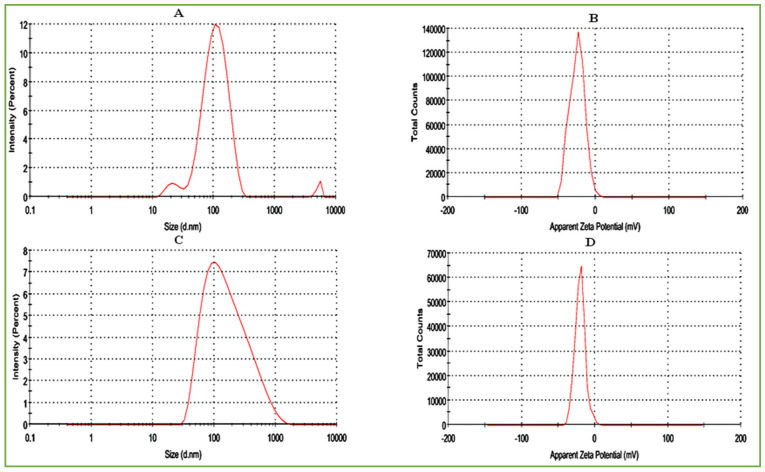
Particle size, size distribution, and zeta potential analysis of AgNPs (**A**,**B**) and Val-AgNPs (**C**,**D**).

The nano-dimensions of AgNPs and Val-AgNPs were confirmed in a suspension under Brownian motion scatter light at different intensities by dynamic light scattering (DLS) analysis. The time-dependent fluctuations in the scattering intensities of AgNPs are converted to a size and size distribution using the Stokes–Einstein relationship. In Figure 7A, the AgNPs showed three levels of particle distribution (diameter in nanometers nm), along with a percentage intensity possessing 116.6 nm (93.6%) and 22.69 nm (4.6%), and an aggregated lump of particles of 5232 nm (1.8%), respectively. However, the Z-average (d.nm) hydrodynamic diameter was 93.46 with a polydispersity index (Pdi) of 0.268, measuring the relatively uniform heterogeneity of AgNPs in a suspended sample. PDI < 0.3 indicates a homogeneous population of AgNPs [40]. For the zeta potential analysis, an electric field is applied to AgNPs in solution and a potential is built up at the slipping plane within the particle’s electric double layer. The Zeta potential, 24.7 (mV), conductivity of 3.14 (mS/cm), and count rate of 140,000 (Figure 7B) was recorded for the particles and showed the highest percentage intensity and Z-average (93.46 d.nm) hydrodynamic diameter (Figure 7A). The Val-AgNPs showed one level of particle size distribution by intensity along with an increased size (208.8 d.nm). In addition, an increase in the hydrodynamic diameter, i.e., a 112.9 Z-average and 0.4 polydispersity index, was observed. However, a two-fold decrease in the percentage intensity was observed in Figure 7C as compared to AgNPs (Figure 7A). In addition, a two-fold decrease in the count rate, i.e., 60,000, zeta potential −19.5 (mV), and conductivity 0.0520 (mS/cm) for Val-AgNPs was recorded (Figure 7D). An increase in the particle size and polydispersity index (broadness of the molecular weight distribution) and decrease in the zeta potential and conductivity in Val-AgNPs might result due to the conjugation of hydrophobic valsartan onto the surface of the silver nanoparticle, which lead to a significant variation in the particle sizes [38]. 

### 2.6. Differential Scanning Calorimetery

**Figure 8:**
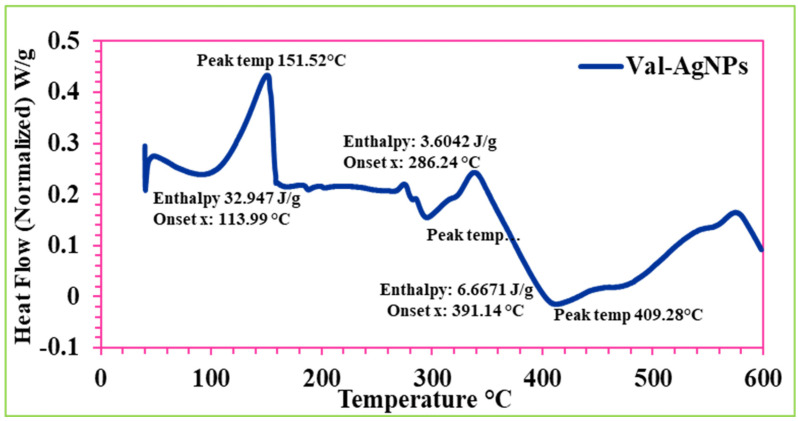
Differential scanning calorimetery presents the thermal analysis of Val-AgNPs.

The thermogram in Figure 8 displayed the thermal behavior of Val-AgNPs, revealing three distinct onset and endset temperatures in the DSC analysis by Ghanbari et al., 2023 (Figure 8) [41]. The first temperature, 113.99 °C, primarily indicates the melting onset (T_onset) of valsartan followed by the peak temperature of 151.03 °C and endset temperature (T_endset) of 158.53 °C, where a thermal event (melting) finishes, marked by the point where the curve returns to the baseline or a new stable state, while the enthalpy of the fusion of Val-AgNPs was calculated as 32.947 J/g (Figure 8). A second T_onset occurred at 286.24 °C followed by a peak temperature of 295.71 °C and T_endset at 332.74 °C with an enthalpy of 3.6042 J/g. This demonstrates the size-dependent densification (melting) of AgNPs in the 40–60 nm range, showing a significant depression from bulk Ag’s 961 °C to a start around 286 °C, peaking at 295.71 °C, with significant surface energy effects (enthalpy of 3.6042 J/g) and densification/sintering occurring up to 332.74 °C, typical for nanostructured materials where a high surface area leads to lower melting points and sintering [3,42,43,44,45]. The third exothermic peak at 391.14 °C, peak temperature of 409.28 °C, and T_endset of 448.08 °C with an enthalpy of normalization 6.6671 J/g may correspond to show the melting temperature of bulk silver nanoparticles that may reach up to 600 °C [46].

Studies show variations in the T_onset, peak temperature, and T_endset of pure valsartan. In a recent study, pure valsartan showed a T_onset of 96.76 °C, a peak temperature of 101.24 °C, and T_endset of 104.89 °C in the DSC analysis [47]. However, Skotnicki et al., 2015 [45] reported that the standard DSC curves of valsartan showed two endothermic events—one at around 60–90 °C (ΔH  =  5  ±  1 J g^−1^) corresponding to a loss of water/adsorbed solvent, and a second event with onset at 98.2  ±  0.9 °C (ΔH VAL  =  26  ±  2 J g^−1^) corresponding to an enthalpy relaxation peak overlapped with a change in the heat capacity. Sabry and coworkers (2023) [40] showed that the peak temperature of valsartan was 101.62 °C and a substantial decrease in the peak temperature (69.1 °C) was recorded for the valsartan-loaded solid lipid nanoparticles. Similarly, in another study, the DSC thermogram of commercial valsartan with crystalline acicular demonstrated a melting peak at 83 °C with an enthalpy value of 26.98 J/g. However, the nanostructured valsartan microparticles showed a decrease in the melting temperatures and enthalpy reduction [3].

### 2.7. HPTLC and HPLC Analysis

**Figure 9:**
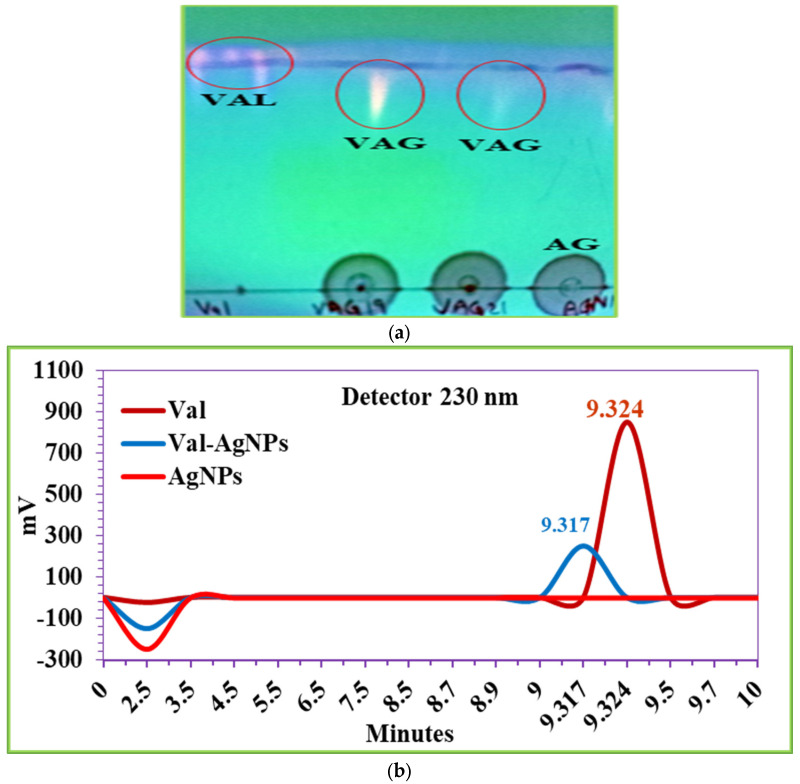
(**a**) HPTLC analysis indicates the illuminated spots of valsartan (VAL) and Val-AgNPs (VAG) at 254 nm and differential migration rates. While no fluorescence and migration along the solvent phase is evident for AgNPs (AG). (**b**) Reverse-phase HPLC chromatograms showing the separation profiles of pure valsartan and Val-AgNPs, respectively.

The HPTLC chromatogram in Figure 9a shows the separation of valsartan and valsartan silver nano-conjugates (Val-AgNPs) in a newly optimized solvent system consisting of methanol, acetic acid, chloroform, and ethyl acetate 5:5:3:3 (*v*/*v*). The calculated retention factors (Rf) of valsartan and Val-AgNPs were 0.92 and 0.746, respectively. The chromatogram displays the UV-illuminated spots on TLC plates which confirm the presence of valsartan in nano-conjugates (VAG) [48]. However, there was no florescence observed in the spot with AgNPs (AG). In previous reports, the HPTLC approach was utilized for a quantitative analysis of valsartan in tables using solvent systems including dichloroethane: methanol: triethylamine (4.2:0.4:0.4 *v*/*v*/*v*) and chloroform: acetonitrile: toluene: glacial acetic acid, in the ratio of 1:8:1:0.1 (*v*/*v*) (*v*/*v*) as the mobile phase, and the retention factor of valsartan was 0.65 [49,50]. In another study, the mobile phase consisting of chloroform:methanol:toluene:glacial acetic acid (6:2:1:0.1 *v*/*v*/*v*/*v*) gave Rf values of 0.36 for valsartan [51].

The chromatogram developed through reverse-phase HPLC (RP-HPLC) showed the separation profile of valsartan and Val-AgNPs (Figure 9b). These results indicate a slight change in the retention time between standard valsartan and Val-AgNPs nanoparticles. This slight decrease in the retention time might result in the decrease in the hydrophobicity (greater polarity) of the silver nano-conjugates of valsartan as compared to valsartan itself. However, the AgNPs did not show interactions with the reverse-phase column [52,53].

### 2.8. pH Stability

**Figure 10:**
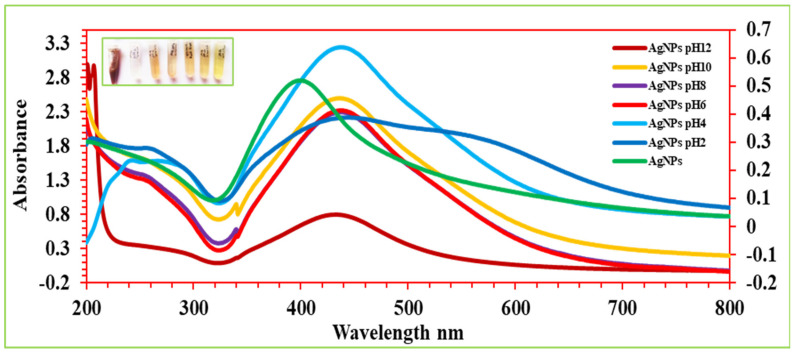
UV-Vis spectrogram showing the effect of pH variability on the stability of biosynthesized AgNPs.

**Figure 11:**
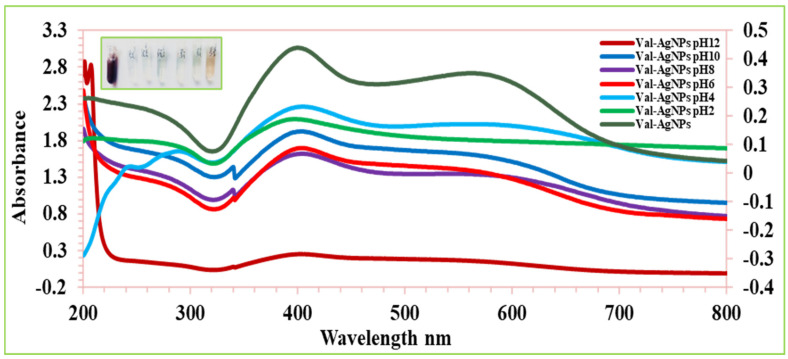
The effect of pH variability on the stability of biosynthesized Val-AgNPs.

The pH of the reaction contents has a significant impact on the biosynthesis and stability of nonomaterials because it influences the electrical charges of biomolecules, changing their reducing and capping abilities [25,54]. The influence of the pH on the SPR band and stability of AgNPs and Val-AgNPs was investigated by changing the pH in a range of 2–12 (Figure 10 and Figure 11). The AgNPs showed the perturbation of the SPR and hypochromic effect and absorption of a longer wave length (bacthochormic shifts), increased by 15–20 nm along with an increase in the area under the curve (peak broading) when AgNPs are suspended in pH solutions of 2, 6, 8, 10, and 12. An increase in the hypochromic effect was observed in the order of the pH range of 10, 6, and 8 and 2 and 12. The noticed hypochromic effect and bathochromic shift (toward longer wavelengths) of the SPR might result due to oxidation Ag^0^ to Ag^+^ and a decrease in the distance between nanoparticles produces the aggregation of the nanoparticles, inducing a strong plasmon coupling between nearby nanoparticles [55]. In contrast, the AgNPs suspended in a pH 4 solution showed an increased SPR intensity. The particles in the pH 6, 8, and 10 solution showed the most stable AgNPs, where no significant shift in the maximum of the SPR band was observed. However, the extreme acidic and alkaline environment results in potential aggreation at pH 2 (peak broadening in the range of 300–800 nm) and the substantial oxidation of Ag^0^ to Ag^+^ complemented by hyochromic SRP and the appearance of the absorption maxima at 405 nm of the silver nitrate. The Val-AgNPs showed SPR perturbation, a hypochromic effect, and bacthochormic shifts when suspended in pH solutions of 2, 6, 8, 10, and 12. The hypochromic effect of Val-AgNPs was observed in the order of pH 10 followed by pH 6, 8, 2, and 12. However, at pH 6–10, the valsartan silver nanaocongugates showed a stable SRP (Figure 11) [56,57].

### 2.9. Effect of MeOH on the Stability of Val-AgNPs

**Figure 12:**
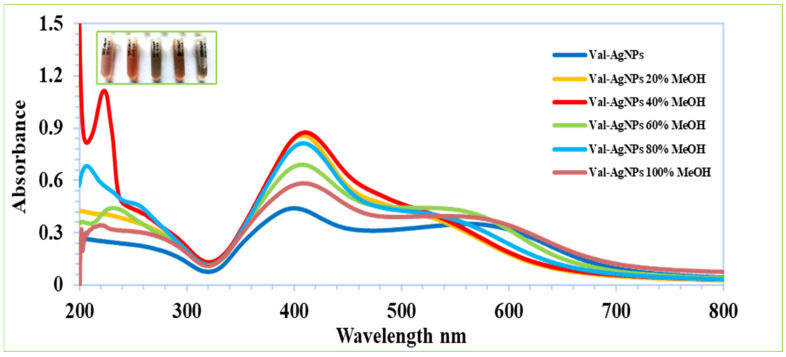
Plasmon resonance spectra of Val-AgNPs in different % solutions of MeOH.

The solvent environment is of great importance because the solubility of a drug varies with the change in the solvent composition. The effect of methanol concentrations, i.e., 20–100% (*v*/*v*), on the stability and surface plasma resonance of Val-AgNPs is illustrated in Figure 12. These results showcase the increase in the SPR intensities followed in the order of the percentage methanol solution: 100% > 60% > 80% > 20% > 40% [58,59]. The most stable SPR was observed in the 100% and 60% methanol solutions (at 540–560 nm), which showed a hypsochromic shift (a shift to shorter wavelengths) upon Val-AgNPs complexation. The off-loading (release) of free valsartan was detected in the 40% and 80% methanolic solutions, identified by the characteristic absorption maximum of free valsartan at 249 nm in its spectral range (Figure 12).

### 2.10. Hemolysis and Effect on Blood Cell Indices (Hemocompatibility)

**Figure 13:**
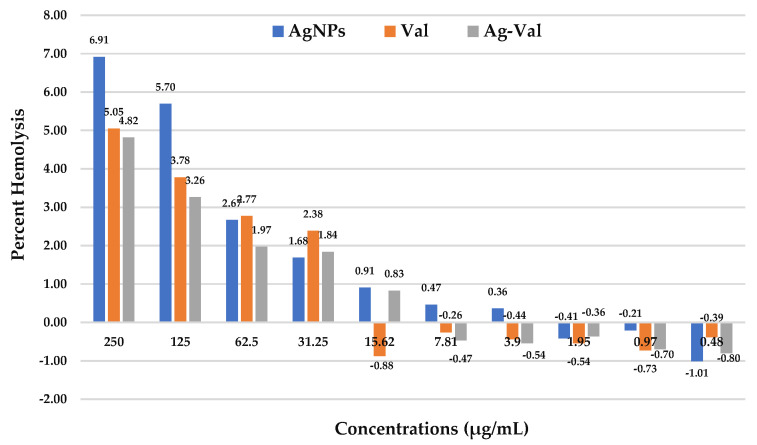
Comparative analysis of Human RBC lysis (%) induced by AgNPs, Val-AgNPs, and valsartan in washed RBC after 3 h at concentrations ranging from 0.48 to 250 μg/mL.

Nanomaterials are, to some degree, incompatible with blood because they can either disrupt the blood cells (hemolysis) or affect the blood cell indices. This incompatibility, or hemotoxicity, is a major concern for their biomedical application and depends heavily on the nanomaterials’ specific physicochemical properties [60]. AgNPs induce hemolysis (0.36%) at a dose of 3.9 μg/mL followed by a dose-dependent increase in hemolysis (6.91%) (Figure 13). The hemolytic activity of AgNPs is mainly attributed to direct nanoparticles–cellular interactions where the particles bind to thiol groups of biological moieties such as proteins and phospholipids in the erythrocyte membrane, leading to denaturation and impaired membrane functioning [61]. Additionally, the negative charge on surface functionalized AgNPs will have strong interactions with biological cations in the erythrocyte membrane, further contributing to hemolysis [60,62]. However, valsartan-induced hemolysis (2–5 percent) was observed at a dose of 31.5 to 250 μg/mL (Figure 13). It is reported that antihypertensive medication with angiotensin-converting enzyme inhibitors can be associated with a reduction in the hemoglobin concentration as a result of hemodilution, hemolytic anemia, and the suppression of red blood cell production [63]. In preclinical safety studies, high doses of valsartan (200 to 600 mg/kg/day body weight) caused in rats a reduction in the red blood cell parameters (erythrocytes, hemoglobin, and hematocrit). In controlled clinical trials, greater than 20% decreases in hemoglobin and hematocrit were observed in 0.4% and 0.8%, respectively, of patients treated with valsartan compared with 0.1% and 0.1% of patients given placebo [64]. The Val-AgNPs showed hemolytic behavior at 15.2 μg/mL, which approaches its maximum hemolysis of 4.82% at its highest dosage (Figure 13). Interestingly, the nano-conjugation of valsartan with silver nanoparticles (Val-AgNPs) decreases the percentage of hemolysis as compared to AgNPs and Val. These observations suggest that while both silver nanoparticles (AgNPs) and valsartan alone induce a notable level of hemolysis, their nano-conjugation into Val-AgNPs significantly decreases the percentage of hemolysis, making the combined form potentially less harmful to red blood cells. Table 2 shows the hemocompatibility of AgNPs, Val-AgNPs, and valsartan assessed after three hours of incubation with whole blood at the highest concentrations (250 μg/mL). The complete blood count did show little variations in the total counts of red cells and hematocrit in the sample incubated with AgNPs (250 μg/mL); however, these indices are not affected by Val-AgNPs and valsartan. A slight decrease in the total platelet count was observed in valsartan and Val-AgNPs (Table 2). On the other hand, there is a slight increase in the percentage of lymphocytes and an increased percentage of the neutrophils count observed as compared to the control; however, the count lies in a normal reference range (Table 2).

## 3. Discussion

Hypertension is a major cardiovascular risk factor, contributing substantially to the global burden of cardiovascular disease and related disability. Despite this, effective blood pressure control remains a largely unmet challenge for public health systems worldwide. One of the key challenges in managing hypertension is the low bioavailability of many antihypertensive drugs, which compromises therapeutic effectiveness. Since only a limited portion of the administered dose enters systemic circulation, higher doses are often required to achieve adequate blood pressure control. This can increase the risk of adverse effects and contribute to poor patient adherence to treatment regimens [65].

Valsartan, N-[p-(o-1H-tetrazol-5-ylphenyl) benzyl]-N-valeryl-l-valine, is an effective long-acting non-peptide AII type 1 receptor antagonist. Valsartan is put forward to treat hypertension, which is one of the leading problems. Furthermore, valsartan blocks the angiotensin receptor, hence delaying the coupling of the angiotensin II receptor, which normalizes blood pressure. Arteries and the heart may witness structural changes caused by angiotensin II receptors [66]. However, valsartan finds poor bioavailability while being orally administered. This drug is partially insoluble in water (0.021 mg mL^−1^), but dissolves quite easily in organic solvent like methanol and ethanol. The insolubility of drugs in water has been a major hurdle in the pharmaceutical formulation, involving preparation stability and drug bioavailability. To enhance the dissolution, the amendment of solid phases is carried out to lower the lattice energy or to break up hydrogen bonding between water molecules by some modification. Dissolution and gastrointestinal permeability are those parameters which control the rate and extent of drug absorption and its bioavailability [67,68].

Nanotechnology has brought enormous advancements, particularly in the field of nanomedicine for enhanced drug delivery using nanoformulations. So far, studies on the development of solid lipid nanoparticles and polymeric nanoparticles aim to transport valsartan across the blood–brain barrier, enhancing intestinal absorption and oral bioavailability, which provide better ways for hypertension management and to mitigate the adverse effects of [3,40,47]. Interestingly, a recent advancement has been made by Katamesh and coworkers in 2026 to enhance skin regeneration using a combination of valsartan-loaded spanlastics gel (Val-SP-gel) and cold atmospheric plasma (CAP), leveraging their anti-inflammatory and regenerative properties. The silver nanoparticles are widely used in biomedical applications. However, little is known about the silver nano-conjugates’ antihypertensive formulations. The precise combination for dermal hypertension management appears to be a topic of experimental inquiry rather than an established clinical treatment [69].

The primary goal of this study was to formulate stable valsartan silver nano-conjugates (Val-AgNPs) through bio-reduction using *Mangifera indica* leaf extracts, a rich source of phytochemicals that reduces silver into stabilized globular nano-conjugates, demonstrating the enhanced solubility of valsartan [70,71]. In addition, Val-AgNPs might be used for dermal application in the management of hypertension. The formation of silver nanoparticles (AgNPs) and valsartan-loaded silver nanoparticles (Val-AgNPs) was visualized by a noticeable color change and specific SRPs observed at 400 nm, 402 nm, and 562 nm. The change in the localized surface plasmon resonance (LSPR) band (the 562 nm peak) confirms that the electronic environment around the silver nanoparticles has changed due to the complexation or binding of the valsartan molecules to the surface of the AgNPs. This binding alters the electron density and the dielectric constant surrounding the nanoparticles, which in turn changes the frequency at which the surface electrons resonate with light. Therefore, spectral data are strong evidence for the successful synthesis and functionalization of the silver nanoparticles with valsartan. In addition, the valsartan coating acts as a surface-modifying agent that alters both the electronic environment of the silver nanoparticles, changing their interaction with UV and visible light, and their surface chemistry, making them hydrophobic on the glass surface in contrast to AgNPs. The conjugation of valsartan with metallic silver is ideally characterized through the functional groups via the IR spectrum. Valsartan-functionalized silver nanoparticles (Val-AgNPs) show an increase in the percentage absorbance of IR as compared to the pure valsartan peaks.

The observed spectral shifts in the Val-AgNPs spectrum compared to pure valsartan indicate molecular interactions (such as binding, capping, or stabilization) between the valsartan functional groups and the silver nanoparticles (AgNPs). These shifts confirm that specific parts of the valsartan molecule are involved in the process of binding to the AgNPs surface. The peak at 1606 cm^−1^ was specifically noted as a significant amide peak used for baseline correction and analysis in the pure valsartan sample. The electron micrographs show that the Val-AgNPs are globular in shape and present in large quantities. The average size for singlet particles is in the range of 30 to 40 nm, with various multimeric forms (di, tri, tetra, and octameric) also observed. Agglomeration likely occurred due to the sample preparation (drying) or the hydrophobic interactions of valsartan, leading to a larger surface-area-to-volume ratio that promotes aggregation to reduce surface energy. In addition, the log-normal distribution of valsartan-loaded nanoparticles, with a size range of 30 to 60 nm and a mode of 54 nm, indicates a narrow, monodisperse, and highly uniform particle size distribution. This is a favorable characteristic for drug delivery systems, as it leads to enhanced bioavailability and a consistent performance. The agglomeration properties of Val-AgNPs are also complemented by DLS and zeta potential analysis. The conjugation of a hydrophobic drug like valsartan onto the surface of silver nanoparticles (Val-AgNPs) can lead to the observed changes in physical properties: an increased particle size, increased polydispersity index (PDI), and a decrease in the zeta potential and conductivity.

Differential Scanning Calorimetry (DSC) is a technique used to measure the heat flow associated with material phase transitions, such as melting. The thermal decomposition of Val-AgNPs observed in this study was 113.99 °C to 158.53 °C, a peak at 151.03 °C, and an enthalpy of fusion of 32.947 J/g. However, the size-dependent densification and melting/sintering of the silver nanoparticles themselves (specifically those in the 40–60 nm range) are characteristic of nanomaterials with high surface energy. The peak was observed at 295.71 °C with an enthalpy of 3.6042 J/g. The 40–60 nm silver nanoparticles melted at a temperature several hundred degrees lower than the bulk material due to their large surface-area-to-volume ratio and lower coordination of surface atoms, which requires less energy to transition to a liquid state. The observation that Val-AgNPs have a slightly decreased retention time compared to pure valsartan supports the hypothesis that the val-AgNPs conjugate is slightly more polar (less hydrophobic) than the un-conjugated valsartan molecule itself. This change in polarity is likely due to the conjugation process involving the AgNPs. The fact that the AgNPs did not show interactions with the reverse-phase column suggests that the silver nanoparticles alone are highly polar or insoluble in the mobile phase and are likely flushed through quickly, confirming that the change in the retention time is linked to the modified valsartan structure rather than the AgNPs themselves interacting with the column matrix in the reverse-phase mode of HPTLC and HPLC. In addition, the retention factor value of valsartan (0.92) is higher than that of Val-AgNPs (0.746), indicating that valsartan travels further up the TLC plate than the Val-AgNPs. This difference in Rf values is significant and confirms that the conjugation of valsartan to silver nanoparticles (Val-AgNPs) changes its physicochemical properties, likely increasing its polarity or interaction with the polar stationary phase.

Dissolution is a complex phenomenon that can be influenced by the factors including the temperature, molecular structure of the drug and solvent, molecular size, etc. In this study, the stability and offloading behavior of Val-AgNPs was observed at pH 6–10 and in 40% and 80% MeOH.

The nano-conjugation of valsartan with silver nanoparticles (Val-AgNPs) significantly decreases the percentage of hemolysis compared to silver nanoparticles (AgNPs) and valsartan (Val) might result in the surface modification of the nanoparticle. The reduced toxicity is likely due to the valsartan coating on the AgNPs, which improves the nanoparticle’s stability and biocompatibility with red blood cells (RBCs). This finding is highly significant for the potential biomedical application of Val-AgNPs. Improving the hemocompatibility of AgNPs is a major goal in nanomedicine, as it determines their suitability for intravenous use or in blood-contacting medical devices. By reducing the harmful effects on red blood cells, the nano-conjugation makes the combined form a promising candidate for further therapeutic development with an improved safety profile.

## 4. Materials and Methods

### 4.1. Chemicals and Plant Material

All the chemicals used in this study were of analytical and HPLC grade. Silver nitrate (AgNO_3_), sodium hydroxide (NaOH), potassium bromide (KBr), sodium chloride (NaCl), methanol (CH_3_OH), glacial acetic acid (CH_3_COOH), chloroform (CHCl_3_), ethyl acetate (C_2_H_5_CH_3_COO^−^), and hydrochloric acid (HCl) were purchased from Merck (Darmstadt, Germany). An active pharmaceutical ingredient (API) of Valsartan (Val), N-[p-(o-1H-tetrazol-5-ylphenyl) benzyl]-N-valeryl-l-valine, was obtained from PharmEvo pharmaceuticals, Karachi, Pakistan. Milli-Q^®^ ultrapure water (18.2 MΩ·cm; IQ 7000 Ultrapure Water Purification System, Merck KGaA, Darmstadt, Germany) was used for the solution preparation and dispersion of nanoparticle. The fresh mango (*Mangifera indica*) leaf samples were collected from the premises of the Department of Biochemistry, University of Karachi in the month of December 2023.

### 4.2. Preparation of Mangifera indica Leaf Extract

The leaves of *Mangifera indica* were washed completely with running tap water to eliminate the dust and particulate matter. Furthermore, the leaves were sterilized with methanol (70% *v*/*v*), then washed thrice with deionized (DI) water and dried on the water-absorbent paper at room temperature [32,72]. For aqueous extract preparation, 0.33 g leaves were homogenized into 5 mL DI water using POLYTRON PT-2100 bench top homogenizer (Kinematica AG, Littau-Lucerne, Switzerland) at room temperature. The extracts were centrifuged at 5000 rpm using Labofuge 200 centrifuge (Heraeus, Hanau, Germany) and filtered out using Whatman no.1 filter paper (Merck KGaA, Darmstadt, Germany) into a clean and dry sterilized laboratory reagent bottle. The prepared green extract solution was stored in the refrigerator for further use [73].

### 4.3. Green Synthesis of Silver Nanoparticles (AgNPs)

For the reductive biosynthesis of silver nanoparticles (AgNPs), a 0.0057 M solution of AgNO_3_ was prepared in deionized water and used as a precursor for silver ions. Around 20 mL of precursor solution was added drop-wise with the *Mangifera indica* leaves extract in a sterilized airtight screw-cap glass vial for the reduction of silver with constant stirring using magnetic stirrer (STUART Scientific, Stone, Staffordshire, United Kingdom) at room temperature. Reduction was performed in a dark environment by covering the vial with aluminum foil [74]. The change in color of the solution from colorless to light yellow then reddish brown indicates the formation of silver nanoparticles. The solution was kept overnight under continuous stirring for the complete reduction of Ag^+^ to Ag^0^ and stabilized to obtain monodispersed nanoparticles [75].

### 4.4. Green Synthesis of Valsartan Silver Nano-Conjugates (Val-AgNPs)

Valsartan solution (Val, 0.0228 M) was prepared by dissolving valsartan into a methanol and DI water (1:4 *v*/*v*) solution in a screw-cap glass vial. The solution was kept for continuous stirring on a magnetic hotplate at room temperature. To this freshly prepared 0.0057 M, AgNO_3_ solution was added dropwise with constant stirring [76]. Valsartan silver nano-conjugates (Val-AgNPs) were prepared through reductive biosynthesis following the dropwise addition of leaf extract (reductant and capping agent) to valsartan and AgNO_3_ solution. The drug, silver nitrate, and leaf extract were mixed in a ratio order of 1:2:2. The glass vial was covered with aluminum foil and kept overnight with continued stirring at room temperature to obtain stabilized Val-AgNPs [77].

### 4.5. Characterization Techniques

Standard characterization techniques including UV-Vis and FTIR spectroscopy, microscopy (SEM-EDS and AFM), and scattering analysis was performed to determine their spectral properties, functional groups, size, and morphology of nanoparticles. Zeta potential measurements and thermal analysis through differential scanning calorimetry (DSC) were performed to determine the surface properties, surface area, and thermal stability, respectively.

#### 4.5.1. Spectrophotometric Characterization of *Mangifera indica* Leaf Extract, AgNPs, and Val-AgNPs

The absorption spectrum of *Mangifera indica* leaf extract, silver nitrate, valsartan, AgNPs, and Val-AgNPs was determined using quartz cuvettes and a Shimadzu UV-1800 double beam UV-Vis spectrophotometer (Shimadzu Corporation, Kyoto, Japan) with spectral bandwidth of 1 nm and wavelength accuracy ±0.5 nm. The AgNPs and Val-AgNPs were aggregated through high-speed centrifugation at 14,000 rpm for 15 min at 25 °C using Eppendorf centrifuge 5415R, Germany. The nanoparticles were washed thrice followed by successive centrifugation to remove remaining reactants. The AgNPs and Val-AgNPs were dispersed in DI water through continuous ultrasonic impulses of 10 watts (RMS) produced by Microson ultrasonic liquid processor MISONIX XL 2000 (Misonix, Inc., Newtown, CT, USA). The surface plasma resonance peaks were recorded between 200 nm and 800 nm. The spectrum was analyzed and annotated with UV-Probe v2.62 software [78].

#### 4.5.2. FTIR Characterization

The characteristic functional groups of valsartan were fingerprinted onto Val-AgNPs through the Shimadzu IRPrestige-21 FTIR spectrophotometer (Shimadzu Corporation, Kyoto, Japan). Briefly, the washed AgNPs, Val-AgNPs, and KBr were oven-dried overnight at 40 °C to remove moisture. The dried nanoparticles and API valsartan were embedded in a KBr matrix separately to produce pellets. The valsartan pallet was taken as a standard and the functional groups were characterized in the range of 400–4000 cm^−1^ [79].

#### 4.5.3. Scanning Electron Microscopy and Energy-Dispersive Spectroscopy

The morphological dimensions, particle size, and distribution of Val-AgNPs were evaluated after gold coating in a vacuum sputter coater Sc7620, (Quorum Technologies Ltd., Laughton, East Sussex, United Kingdom) and micrographs were obtained via scanning electron microscope Apreo 2C, scanning electron microscope, (Theromo Scientific, Waltham, MA, USA) at scale of 100 and 400 nanometers, and resolved at 200,000×, 450,000×, and 600,000×, magnification using accelerated electron beam of 30.0 KV, optimized to obtain homogenous scans. The energy-dispersive spectroscopy (EDS) was performed using the air-dried Val-AgNPs. The mapping of elements was performed at 500 nanometers, and resolved at 120,000×, using an accelerated electron beam of 30.0 KV. The elemental analysis was performed and total number of counts was recorded during 30 s acquisition time [9,80]. In solution (dispersed nanoparticles in DI water), EDS profile was complimented through JEOL-JSM-6380A scanning electron microscope (JEOL Ltd., Akishima, Tokyo, Japan).

#### 4.5.4. Atomic Force Spectroscopy

High-resolution atomic force microscopic three-dimensional imaging was performed to evaluate nanoscale details of particle shape, topographical map, distribution of particle sizes, height, volume, and surface texture. For immobilization, the nanoparticles were dispersed in DI and spotted on poly-L-lysine-coated mica slides and air-dried at room temperature prior to AFM analysis. Morphological changes were studied with an atomic force microscope Agilent 5500 (Agilent Technologies, Santa Clara, California, United States) via tapping mode using silicon nitride cantilever (Veeco, model MLCT-AUHW) and a spring constant value of 0.1 Nm^−1^ was utilized with a resonating frequency of 323.673 kHz [81]. Images were captured at an optimized scan velocity of 1–5 μm/s and 512 × 512-line resolution and processed through PicoView 1.2 imaging software.

#### 4.5.5. Zeta Potential Measurements and Particle Size Analysis

Zeta potential measurements and particle size distribution of AgNPs and Val-AgNPs were investigated using the Malvern Instruments Nano-ZSP zeta sizer (Malvern Panalytical, Malvern, United Kingdom). The analysis was conducted at a temperature of 25 °C, with a constant scattering angle of 90°. Particle size measurements were performed using a disposable cuvette, while zeta potential determinations utilized a cell immersed in a disposable cuvette [82].

#### 4.5.6. Differential Scanning Calorimetry

DSC analysis of Val-AgNPs was performed using a TA Instruments Trios V5.3.0.48151 Differential Scanning Calorimeter (TA Instruments, New Castle, DE, USA). The Val-AgNPs were weighed and put in aluminum hermetic pans and were crimped, followed by heating in an inert atmosphere maintained by purging nitrogen at a flow rate of 50 mL/min. Heating was set to run from 20 to 600 °C at a flow rate of 10 °C per minute for each sample. An empty pan was used as a reference. The heat flow as a function of temperature was determined for drug-loaded nanoparticles [83].

### 4.6. HPTLC and HPLC Analysis

High-performance thin-layer chromatographic (HPTLC) is an analytical method used to identify the presence of compounds in non-volatile mixtures. HPTLC analysis of valsartan and the detection of valsartan on Val-AgNPs was performed on a sorbent silica gel plate, Silica Gel 60 F_254_, (Merck KGaA, Darmstadt, Germany). The solution containing 2 µg/µL AgNPs, Val-AgNPs, and valsartan were applied in spots (5 µg) and air-dried. The plates were developed with methanol, acetic acid, chloroform, and ethyl acetate 5:5:3:3 (*v*/*v*) as mobile phase [84]. The chromatographic plate was dried at room temperature and the presence of standard valsartan and valsartan silver nano-conjugates was detected at 254 nm using the UV transilluminator FOTO/UV-21 (Fototdyne, Hartland, WI, USA).

High-performance liquid chromatographic analysis (HPLC) profiling of Val-AgNPs involves separation and identification of valsartan, in the form of Val-AgNPs complex. Analysis was performed using a reverse-phase C18 (4.6 mm × 150 mm I.D, CS-ODs 100-5) µm column (Merck KGaA, Darmstadt, Germany). Conjugated valsartan (Val-AgNPs) and standard were prepared at a concentration of 20 mcg in acetonitrile + water (50:50 *v*/*v*) in a flask. The samples were sonicated and filtered using a 0.22 µ syringe filter (Merck, Millipore, Darmstadt, Germany) and injected into a column. The mobile phase used for the development of chromatogram was composed of a mixture of water, acetonitrile, and glacial acetic acid (50:50:0.1 *v*/*v*) and delivered at a flow rate of 1 mL/min at room temperature. The detection of valsartan was conducted at a specific, optimal wavelength of 230 nm, chosen within the broader spectral range of the detector’s capability (200–400 nm) [85,86].

### 4.7. pH Stability

The pH and solvent environment are among key parameters in the synthesis, stability, and solubility of the nanocomplexes. The colloidal stability of synthesized Val-AgNPs was screened at different pHs. The Val-AgNPs were dispersed in different pH solutions adjusted with the help of 0.1M HCl and 0.1M NaOH [11]. The stability and solubility of Val-AgNPs was studied in 100%, 80%, 60%, 40%, and 20% methanol water (*v*/*v*) solutions [58,59,86].

### 4.8. Hemolysis and Effect on Blood Cell Indices (Hemocompatibility)

The hemolytic activity of valsartan, AgNPs, and Val-AgNPs was determined by the release of hemoglobin from the erythrocyte cells during their incubation under physiological conditions. Minimizing the confounding factors, blood samples were drawn in tubes with ethylenediaminetetraacetic acid (anticoagulant) from healthy volunteers potentially refrain from any drug for 24 h to ensure the accuracy of the assay. Phosphate buffer solution (PBS, pH 7.4)-washed erythrocytes were prepared through centrifugation using Labofuge 200, (Heraeus, Hanau, Germany) at 2500 rpm for 10 min. Different concentrations of the valsartan, AgNPs, and Val-AgNPs (250, 125, 62.5, 31.25, 15.62, and 7.81 µg/mL) were formulated in PBS and incubated with 100 µL of 10% washed red blood cells. A 10% triton X-100 (Merck KGaA, Darmstadt, Germany) was used as a positive control, while only PBS was used instead of the samples as a negative control. After an incubation period of three hours at 37 °C, the samples were centrifuged at 3000 rpm for 10 min. Then, the absorbance of the supernatant was measured at 540 nm using a BioBase microplate reader (BIOBASE Group, Jinan, Shandong, China). The average percentage of hemolysis was calculated using the following equation: (sample absorbance–negative control absorbance)/(positive control absorbance–negative control absorbance) × 100. The valsartan-, AgNPs-, and Val-AgNPs-induced alterations in hematological indices and cellular morphology was observed via Zybio Z3 hematology analyzer (Zybio Inc., Chongqing, China) and the results were compared with the control blood samples.

## 5. Conclusions

This study concludes that the 30 to 60 nm silver nano-congugates of valsartan (Val-AgNPs) exhibit favorable characteristics for drug delivery systems, as they may lead to enhanced bioavailability and a consistent performance of lipophilic valsartan due to an increase in the polarity, stability, and biocompatabilty. The Val-AgNPs did not show toxic effects or significant alterations in the blood cell indices and were considered nonhemolytic even at a higher concentration, confirming the safety of the nano-conjugates for biological applications. In conclusion, these findings provide a comprehensive characterization of Val-AgNPs, highlighting their potential for improved drug delivery applications. It also signifies the potential of nano-conjugates to improve the solubility and, presumably, the bioavailability of the antihypertensive drug valsartan.

## Figures and Tables

**Table 1:** Elemental analysis of Val-AgNPs obtained through energy-dispersive spectroscopy.

Element	Atomic %	Atomic % Error	Weight %	Weight % Error
C	50.2	0.3	21.0	0.1
N	14.6	1.1	7.1	0.5
O	16.6	0.8	9.2	0.4
Na	0.0	0.0	0.0	0.0
Mg	1.5	0.1	1.2	0.1
Al	0.7	0.0	0.7	0.0
Cl	0.1	0.0	0.1	0.0
Ca	0.1	0.0	0.2	0.0
As	0.1	0.0	0.2	0.1
Mo	0.3	0.2	0.9	0.7
Ag	72.2	0.5	89.4	0.6
Cd	0.1	0.1	0.4	0.3

**Table 2:** Hemocompatibility assay of AgNPs, Val-AgNPs, and valsartan indicated by the complete blood count.

Test Description	Control	AgNPs	Val-AgNPs	Val	Reference	Range Unit (s)
RED BLOOD CELLS	4.01	3.95 *	4.08	4	4.50–5.50	×10^6^/μL
HEMOGLOBIN	13.2	13.1	13.5	13.3	13.0–17.0	g/dL
HAEMATOCRIT	35.2	34.7 *	35.9	35.1	40.0–50.0	%
M.C.V.	87.7	87.7	88	87.8	78.0–100.0	fl
M.C.H.	33	33.2	33.1	33.2	27.0–34.0	pg
M.C.H.C.	37.6	37.8	37.7	37.8	31.0–36.0	gm/dL
RDW-CV	13.7	13.4	13.7	13.6	<14.5%	%
PLATELET COUNT	271	269	260 *	263 *	150–400	×10^3^/μL
Mean Plat Volume (MPV)	10.7	10.5	10.5	10.6	7.1–9.5	fl
TOTAL/DIFFERENTIAL W.B.C. IN %						
W.B.C. COUNT	7.8	7.74	7.81	7.9	4.0–11.0	×10^3^/μL
Neutrophils	66	61 *	62 *	60 *	40–75	%
Lymphocytes	27	32 *	31 *	34 *	20–45	%
Monocytes	5	4 *	6 **	3 *	2–8	%
Eosinophils	2	3	1	3	2–4	%
Basophils	0	0	0	0	<1	%

Key: * = Decrease in count; ** Increase in count; Control = Blood samples did not incubate with AgNPs, Val-AgNPs, and valsartan.

## Data Availability

The original contributions presented in this study are included in this article. Further inquiries can be directed to the corresponding authors.

## References

[1] Dhaval M., Makwana J., Sakariya E., Dudhat K. (2020). Drug Nanocrystals: A Comprehensive Review with Current Regulatory Guidelines. Curr. Drug Deliv..

[2] Quodbach J., Preis E., Karkossa F., Winck J., Finke J.H., Steiner D. (2025). Novel Strategies for the Formulation of Poorly Water-Soluble Drug Substances by Different Physical Modification Strategies with a Focus on Peroral Applications. Pharmaceuticals.

[3] Prieto C., Evtoski Z., Pardo-Figuerez M., Hrakovsky J., Lagaron J.M. (2021). Nanostructured Valsartan Microparticles with Enhanced Bioavailability Produced by High-Throughput Electrohydrodynamic Room-Temperature Atomization. Mol. Pharm..

[4] Abada E., Mashraqi A., Modafer Y., Al Abboud M.A., El-Shabasy A. (2024). Review green synthesis of silver nanoparticles by using plant extracts and their antimicrobial activity. Saudi J. Biol. Sci..

[5] AlMohammed H.I., Khalaf A.K., Albalawi A.E., Alanazi A.D., Baharvand P., Moghaddam A., Mahmoudvand H. (2021). Chitosan-Based Nanomaterials as Valuable Sources of Anti-Leishmanial Agents: A Systematic Review. Nanomaterials.

[6] Kanniah P., Chelliah P., Thangapandi J.R., Gnanadhas G., Mahendran V., Robert M. (2021). Green synthesis of antibacterial and cytotoxic silver nanoparticles by Piper nigrum seed extract and development of antibacterial silver based chitosan nanocomposite. Int. J. Biol. Macromol..

[7] Aslam I., Iqbal J., Peerzada S., Afridi M.S.K., Ishtiaq S. (2019). Microscopic investigations and pharmacognostic techniques for the standardization of *Caralluma edulis* (Edgew.) Benth. ex Hook.f. Microsc. Res. Tech..

[8] Mathur P., Jha S., Ramteke S., Jain N.K. (2018). Pharmaceutical aspects of silver nanoparticles. Artif. Cells Nanomed. Biotechnol..

[9] Abdelghany T.M., Al-Rajhi A.M.H., Al Abboud M.A., Alawlaqi M.M., Magdah A.G., Helmy E.A.M., Mabrouk A.S. (2018). Recent advances in green synthesis of silver nanoparticles and their applications: About future directions—A Review. BioNanoScience.

[10] Ahmad A., Wei Y., Syed F., Tahir K., Rehman A.U., Khan A., Ullah S., Yuan Q. (2017). The effects of bacteria-nanoparticles interface on the antibacterial activity of green synthesized silver nanoparticles. Microb. Pathog..

[11] Miranda A., Akpobolokemi T., Chung E., Ren G., Raimi-Abraham B.T. (2022). pH Alteration in Plant-Mediated Green Synthesis and Its Resultant Impact on Antimicrobial Properties of Silver Nanoparticles (AgNPs). Antibiotics.

[12] Nadaroglu H., Onem H., Gungor A.A. (2017). Green synthesis of Ce_2_O_3_ NPs and determination of its antioxidant activity. IET Nanobiotechnology.

[13] Yu Y., Gong Y., Hu B., Ouyang B., Pan A., Liu J., Liu F., Shang X.-L., Yang X.-H., Tu G. (2023). Expert consensus on blood pressure management in critically ill patients. J. Intensive Med..

[14] Schneider M., Stracke F., Hansen S., Schaefer U.F. (2009). Nanoparticles and their interactions with the dermal barrier. Derm. Endocrinol..

[15] Larese F.F., D’AGostin F., Crosera M., Adami G., Renzi N., Bovenzi M., Maina G. (2009). Human skin penetration of silver nanoparticles through intact and damaged skin. Toxicology.

[16] Zeb A., Arif S.T., Malik M., Shah F.A., Din F.U., Qureshi O.S., Lee E.-S., Lee G.-Y., Kim J.-K. (2019). Potential of nanoparticulate carriers for improved drug delivery via skin. J. Pharm. Investig..

[17] Kraeling M.E., Topping V.D., Keltner Z.M., Belgrave K.R., Bailey K.D., Gao X., Yourick J.J. (2018). In vitro percutaneous penetration of silver nanoparticles in pig and human skin. Regul. Toxicol. Pharmacol. RTP.

[18] Taniguchi M., LaRocca C.A., Bernat J.D., Lindsey J.S. (2023). Digital Database of Absorption Spectra of Diverse Flavonoids Enables Structural Comparisons and Quantitative Evaluations. J. Nat. Prod..

[19] Azizi-Khereshki N., Mousavi H.Z., Dogaheh M.G., Farsadrooh M., Alizadeh N., Mohammadi A. (2023). Synthesis of molecularly imprinted polymer as a nanosorbent for dispersive magnetic micro solid-phase extraction and determination of valsartan in biological samples by UV–Vis Spectrophotometry: Isotherm, kinetics, and thermodynamic studies. Spectrochim. Acta Part A Mol. Biomol. Spectrosc..

[20] Quevedo A.C., Guggenheim E., Briffa S.M., Adams J., Lofts S., Kwak M., Lee T.G., Johnston C., Wagner S., Holbrook T.R. (2021). UV-Vis Spectroscopic Characterization of Nanomaterials in Aqueous Media. J. Vis. Exp. JoVE.

[21] Samari F., Salehipoor H., Eftekhar E., Yousefinejad S. (2018). Low-temperature biosynthesis of silver nanoparticles using mango leaf extract: Catalytic effect, antioxidant properties, anticancer activity and application for colorimetric sensing. New J. Chem..

[22] Andresen B.T., Anderson S.D., Yeon J.K., Mireles R. (2017). Valsartan. Reference Module in Biomedical Sciences.

[23] Scroccarello A., Molina-Hernández B., Della Pelle F., Ciancetta J., Ferraro G., Fratini E., Valbonetti L., Copez C.C., Compagnone D. (2021). Effect of phenolic compounds-capped AgNPs on growth inhibition of Aspergillus niger. Colloids Surf. B Biointerfaces.

[24] Khalir W.K.A.W.M., Shameli K., Jazayeri S.D., Othman N.A., Jusoh N.W.C., Hassan N.M. (2020). Biosynthesized Silver Nanoparticles by Aqueous Stem Extract of Entada spiralis and Screening of Their Biomedical Activity. Front. Chem..

[25] Dhaka A., Mali S.C., Sharma S., Trivedi R. (2023). A review on biological synthesis of silver nanopSarticles and their potential applications. Results Chem..

[26] Asif M., Yasmin R., Asif R., Ambreen A., Mustafa M., Umbreen S. (2022). Green Synthesis of Silver Nanoparticles (AgNPs), Structural Characterization, and Their Antibacterial Potential. Dose-Response A Publ. Int. Hormesis Soc..

[27] Rahman A., Sravani G.J., Srividya K., Priyadharshni A.D.R., Narmada A., Sahithi K., Sai T.K., Padmavathi Y. (2020). Development and validation of chemometric assisted FTIR spectroscopic method for simultaneous estimation of valsartan and hydrochlorothiazide in pure and pharmaceutical dosage forms. J. Young Pharm..

[28] Harshou N., Trefi S., Bitar Y. (2022). Fourier transform infrared spectroscopy for quantitative determination of valsartan in bulk materials and in pharmaceutical dosage forms. Bull. Pharm. Sci. Assiut.

[29] Kale K.B., Shinde M.A., Patil R.H., Ottoor D.P. (2022). Exploring the interaction of Valsartan and Valsartan-Zn(ll) complex with DNA by spectroscopic and in silico methods. Spectrochim. Acta Part A Mol. Biomol. Spectrosc..

[30] Aisida S.O., Madubuonu N., Alnasir M.H., Ahmad I., Botha S., Maaza M., Ezema F.I. (2020). Biogenic synthesis of iron oxide nanorods using *Moringa oleifera* leaf extract for antibacterial applications. Appl. Nanosci..

[31] Nasrollahzadeh M., Atarod M., Sajjadi M., Sajadi S.M., Issaabadi Z. (2019). Plant-mediated green synthesis of nanostructures: Mechanisms, characterization, and applications. Interface Science and Technology.

[32] Bhuiyan S.H., Miah M.Y., Paul S.C., Das Aka T., Saha O., Rahaman M., Sharif J.I., Habiba O. (2020). Ashaduzzaman Green synthesis of iron oxide nanoparticle using Carica papaya leaf extract: Application for photocatalytic degradation of remazol yellow RR dye and antibacterial activity. Heliyon.

[33] Srećković N.Z., Nedić Z.P., Monti D.M., D’elia L., Dimitrijević S.B., Mihailović N.R., Stanković J.S.K., Mihailović V.B. (2023). Biosynthesis of Silver Nanoparticles Using *Salvia pratensis* L. Aerial Part and Root Extracts: Bioactivity, Biocompatibility, and Catalytic Potential. Molecules.

[34] Ahmed A., Rauf A., Hemeg H.A., Qureshi M.N., Sharma R., Aljohani A.S.M., Alhumaydhi F.A., Khan I., Alam A., Rahman M. (2022). Green synthesis of gold and silver nanoparticles using *Opuntia dillenii* aqueous extracts: Characterization and their antimicrobial assessment. J. Nanomater..

[35] Sivalingam A.M., Pandian A. (2024). Characterization of silver nanoparticles (AgNPs) synthesized using polyphenolic compounds from *Phyllanthus emblica* L. and their impact on cytotoxicity in human cell lines. Carbohydr. Polym. Technol. Appl..

[36] Hodoroaba V.-D., Hodoroaba V.-D., Unger W.E.S., Shard A.G. (2020). Chapter 4.4—Energy-dispersive X-ray spectroscopy (EDS). Micro and Nano Technologies, Characterization of Nanoparticles.

[37] Grobelny J., DelRio F.W., Pradeep N., Kim D.-I., Hackley V.A., Cook R.F. (2005). Size Measurement of Nanoparticles Using Atomic Force Microscopy: Version 1.1. 2009 Oct. National Cancer Institute’s Nanotechnology Characterization Laboratory Assay Cascade Protocols.

[38] Chicea D., Nicolae-Maranciuc A., Doroshkevich A.S., Chicea L.M., Ozkendir O.M. (2023). Comparative synthesis of silver nanoparticles: Evaluation of chemical reduction procedures, AFM and DLS size analysis. Materials.

[39] Ali S., Perveen S., Ali M., Jiao T., Sharma A.S., Hassan H., Devaraj S., Li H., Chen Q. (2020). Bioinspired morphology-controlled silver nanoparticles for antimicrobial application. Mater. Sci. Eng. C.

[40] Sabry S.A., El Razek A.M.A., Nabil M., Khedr S.M., El-Nahas H.M., Eissa N.G. (2023). Brain-targeted delivery of Valsartan using solid lipid nanoparticles labeled with Rhodamine B; a promising technique for mitigating the negative effects of stroke. Drug Deliv..

[41] Ghanbari E., Picken S.J., van Esch J.H. (2023). Analysis of differential scanning calorimetry (DSC): Determining the transition temperatures, and enthalpy and heat capacity changes in multicomponent systems by analytical model fitting. J. Therm. Anal. Calorim..

[42] Luo S., Zhang X., Huang X., Xu W. (2014). Low-Temperature Sintering of Nanosilver Paste on a Gold Film Surface. High Temp. Mater. Process..

[43] Lee S., Phelan P.E., Taylor R.A., Prasher R., Dai L. (2016). Low-temperature melting of silver nanoparticles in sub-cooled and saturated water. J. Heat Transf..

[44] Asoro M., Damiano J., Ferreira P. (2009). Size effects on the melting temperature of silver nanoparticles: In-situ tem observations. Microsc. Microanal..

[45] Nasr A.M., Moftah F., Abourehab M.A.S., Gad S. (2022). Design, Formulation, and Characterization of Valsartan Nanoethosomes for Improving Their Bioavailability. Pharmaceutics.

[46] Demchenko V., Mamunya Y., Sytnyk I., Iurzhenko M., Krivtsun I., Rybalchenko N., Naumenko K., Artiukh L., Kowalczuk M., Demchenko O. (2025). Fabrication of polylactide composites with silver nanoparticles by sputtering deposition and their antimicrobial and antiviral applications. Polym. Int..

[47] Kalyan G.P., Ranganayakulu B. (2025). Preparation and characterization of valsartan nanoparticles. Int. J. Curr. Trends Pharm. Res..

[48] Meyers C., Meyers D. (2008). Thin-Layer Chromatography. Curr. Protoc. Nucleic Acid Chem..

[49] Deore B.L., Patil A.S., Mali B.J., Patil S., Ratnaparkhi S. (2024). HPTLC APPROACH for simultaneous quantification of valsartan and sacubitril in bulk and tablet formulations. World J. Adv. Res. Rev..

[50] Parambi D.G.T., Mathew M., Ganesan V. (2001). Quantitative analysis of Valsartan in tablets formulations by High Performance Thin-Layer Chromatography. J. Appl. Pharm. Sci..

[51] Shah N.J., Suhagia B.N., Shah R.R., Patel N.M. (2009). HPTLC Method for the Simultaneous Estimation of Valsartan and Hydrochlorothiazide in Tablet Dosage Form. Indian J. Pharm. Sci..

[52] Bertrams J., Müller M.B., Kunz N., Kammerer D.R., Stintzing F.C. (2013). Phenolic compounds as marker compounds for botanical origin determination of German propolis samples based on TLC and TLC-MS. J. Appl. Bot. Food Qual..

[53] Kamireddy T., Sambu P., Lankalapalli P.K., Myneni R.K., Divadari H. (2024). Stability-indicating RP-HPLC method development and validation for the quantification of amlodipine besylate and valsartan tablets in solid oral dosage form. Biomed. Chromatogr. BMC.

[54] Velgosova O., Mačák L., Lisnichuk M., Varga P. (2025). Influence of pH and Temperature on the Synthesis and Stability of Biologically Synthesized AgNPs. Appl. Nano.

[55] Silva-Holguín P.N., Garibay-Alvarado J.A., Reyes-López S.Y. (2024). Silver Nanoparticles: Multifunctional Tool in Environmental Water Remediation. Materials.

[56] Anigol L.B., Charantimath J.S., Gurubasavaraj P.M. (2017). Effect of concentration and pH on the size of silver nanoparticles synthesized by green chemistry. Org. Med. Chem. Int. J..

[57] Liaqat N., Jahan N., Rahman K.U., Anwar T., Qureshi H. (2022). Green synthesized silver nanoparticles: Optimization, characterization, antimicrobial activity, and cytotoxicity study by hemolysis assay. Front. Chem..

[58] Dang T.M.D., Le T.T.T., Fribourg-Blanc E., Dang M.C. (2011). The influence of solvents and surfactants on the preparation of copper nanoparticles by a chemical reduction method. Adv. Nat. Sci. Nanosci. Nanotechnol..

[59] Khoza P.B., Moloto M.J., Sikhwivhilu L.M. (2012). The effect of solvents, acetone, water, and ethanol, on the morphological and optical properties of ZnO nanoparticles prepared by microwave. J. Nanotechnol..

[60] de la Harpe K.M., Kondiah P.P., Choonara Y.E., Marimuthu T., du Toit L.C., Pillay V. (2019). The Hemocompatibility of Nanoparticles: A Review of Cell–Nanoparticle Interactions and Hemostasis. Cells.

[61] Laloy J., Minet V., Alpan L., Mullier F., Beken S., Toussaint O., Lucas S., Dogné J.-M. (2014). Impact of Silver Nanoparticles on Haemolysis, Platelet Function and Coagulation. Nanobiomedicine.

[62] Luna-Vázquez-Gómez R., Arellano-García M.E., Toledano-Magaña Y., García-Ramos J.C., Radilla-Chávez P., Salas-Vargas D.S., Casillas-Figueroa F., Ruiz-Ruiz B., Pestryakov A., Bogdanchikova N. (2022). Bell Shape Curves of Hemolysis Induced by Silver Nanoparticles: Review and Experimental Assay. Nanomaterials.

[63] Sica D.A., Mannino R. (2007). Antihypertensive medications and anemia. J. Clin. Hypertens..

[64] Novartis Pharmaceuticals Canada Inc (2025). DIOVAN (Valsartan) Tablets: Product Monograph Including Patient Medication Information. https://www.novartis.com/ca-en/sites/novartis_ca/files/diovan_pm_20250617_en.pdf.

[65] Parati G., Lombardi C., Pengo M., Bilo G., Ochoa J.E. (2021). Current challenges for hypertension management: From better hypertension diagnosis to improved patients’ adherence and blood pressure control. Int. J. Cardiol..

[66] Shen M., Zheng C., Chen L., Li M., Huang X., He M., Liu C., Lin H., Liao W., Bin J. (2023). LCZ696 (sacubitril/valsartan) inhibits pulmonary hypertension induced right ventricular remodeling by targeting pyruvate dehydrogenase kinase 4. Biomed. Pharmacother..

[67] Shi Y.J., Yang C.G., Qiao W.B., Liu Y.C., Liu S.Y., Dong G.J. (2023). Sacubitril/valsartan attenuates myocardial inflammation, hypertrophy, and fibrosis in rats with heart failure with preserved ejection fraction. Eur. J. Pharmacol..

[68] Katamesh A.A., Ibrahim M., Qelliny M.R., Abu Lila A.S., Subaiea G.M., Hassoun S.M., Abdallah M.H., Ismail M.M., El Sayed M.M., Mostafa M. (2026). Innovative synergy in wound repair: Valsartan-loaded spanlastics gel coupled with cold atmospheric plasma for improved skin recovery. J. Drug Deliv. Sci. Technol..

[69] Xu L., Wang Y.-Y., Huang J., Chen C.-Y., Wang Z.-X., Xie H. (2020). Silver nanoparticles: Synthesis, medical applications and biosafety. Theranostics.

[70] Duman H., Eker F., Akdaşçi E., Witkowska A.M., Bechelany M., Karav S. (2024). Silver Nanoparticles: A Comprehensive Review of Synthesis Methods and Chemical and Physical Properties. Nanomaterials.

[71] Arshad F., Naikoo G.A., Hassan I.U., Chava S.R., El-Tanani M., A Aljabali A., Tambuwala M.M. (2024). Bioinspired and Green Synthesis of Silver Nanoparticles for Medical Applications: A Green Perspective. Appl. Biochem. Biotechnol..

[72] Buarki F., AbuHassan H., Al Hannan F., Henari F.Z. (2022). Green synthesis of iron oxide nanoparticles using hibiscus rosa sinensis flowers and their antibacterial activity. J. Nanotechnol..

[73] Sivakami M., Devi K.R., Renuka R., Thilagavathi T. (2020). Green synthesis of magnetic nanoparticles via Cinnamomum verum bark extract for biological application. J. Environ. Chem. Eng..

[74] Bhusal M., Pathak I., Bhadel A., Shrestha D.K., Sharma K.R. (2024). Synthesis of silver nanoparticles assisted by aqueous root and leaf extracts of Rhus chinensis Mill and its antibacterial activity. Heliyon.

[75] Rodríguez-Félix F., López-Cota A.G., Moreno-Vásquez M.J., Graciano-Verdugo A.Z., Quintero-Reyes I.E., Del-Toro-Sánchez C.L., Tapia-Hernández J.A. (2021). Sustainable-green synthesis of silver nanoparticles using safflower (*Carthamus tinctorius* L.) waste extract and its antibacterial activity. Heliyon.

[76] Sur U.K., Ankamwar B., Karmakar S., Halder A., Das P. (2018). Green synthesis of Silver nanoparticles using the plant extract of Shikakai and Reetha. Mater. Today Proc..

[77] Peleshok K., Piponski M., Ajie E.A., Poliak O., Zarivna N., Denefil O., Logoyda L. (2021). Novel HPLC-UV method for simultaneous determination of valsartan and atenolol in fixed dosage form; Study of green profile assessment. Pharmacia.

[78] Fazlzadeh M., Rahmani K., Zarei A., Abdoallahzadeh H., Nasiri F., Khosravi R. (2017). A novel green synthesis of zero valent iron nanoparticles (NZVI) using three plant extracts and their efficient application for removal of Cr(VI) from aqueous solutions. Adv. Powder Technol..

[79] Amutha S., Sridhar S. (2018). Green synthesis of magnetic iron oxide nanoparticle using leaves of Glycosmis mauritiana and their antibacterial activity against human pathogens. J. Innov. Pharm. Biol. Sci..

[80] Rasheed T., Bilal M., Iqbal H.M., Li C. (2017). Green biosynthesis of silver nanoparticles using leaves extract of Artemisia vulgaris and their potential biomedical applications. Colloids Surf. B Biointerfaces.

[81] Talha A., Raja D.A., Hussain D., Malik M.I. (2024). Gold nanoparticle-based selective and efficient spectrophotometric assay for the insecticide methamidophos. Microchim. Acta.

[82] Abdulnaby H.M., Elkashef I., Ibrahim S., Labeeb A.M. (2024). Synthesis of Silver Nanoparticles with Different Decoration Forms Dispersed in Nematic Liquid Crystal. Egypt. J. Chem..

[83] Shewale S., Undale V., Shelar M., Bhalchim V., Panchal C., Gundecha S. (2022). Development & quantitative analysis of validated stability-indicating analytical method for estimation of valsartan and hydrochlorothiazide by high performance thin layer chromatography. Mater. Today Proc..

[84] Ardiana F., Suciati, Indrayanto G. (2015). Valsartan. Profiles of Drug Substances, Excipients and Related Methodology.

[85] Ragab M.A.A., Galal S.M., Korany M.A., Ahmed A.R. (2018). High performance thin-layer and high performance liquid chromatography coupled with photodiode array and fluorescence detectors for analysis of valsartan and sacubitril in their supramolecular complex with quantitation of sacubitril-related substance in raw material and tablets. J. Chromatogr. Sci..

[86] Marciniak L., Nowak M., Trojanowska A., Tylkowski B., Jastrzab R. (2020). The Effect of pH on the Size of Silver Nanoparticles Obtained in the Reduction Reaction with Citric and Malic Acids. Materials.

